# Fc-Galactosylation of Human Immunoglobulin Gamma Isotypes Improves C1q Binding and Enhances Complement-Dependent Cytotoxicity

**DOI:** 10.3389/fimmu.2017.00646

**Published:** 2017-06-06

**Authors:** Benjamin Peschke, Christian W. Keller, Patrick Weber, Isaak Quast, Jan D. Lünemann

**Affiliations:** ^1^Institute of Experimental Immunology, Laboratory of Neuroinflammation, University of Zurich, Zurich, Switzerland; ^2^Department of Immunology and Pathology, Central Clinical School, Monash University, Melbourne, VIC, Australia; ^3^Department of Neurology, University Hospital Zurich, Zurich, Switzerland

**Keywords:** c1q, complement system proteins, antibodies, monoclonal, glycan, IgG subclasses

## Abstract

Binding of the complement component C1q to the CH2 domain of antigen-bound immunoglobulin gamma (IgG) activates the classical complement pathway and depends on its close proximity to Fc fragments of neighboring antibodies. IgG subclasses contain a highly conserved asparagine 297 (N)-linked biantennary glycan within their CH2 domains, the core structure of which can be extended with terminal galactose and sialic acid residues. To investigate whether Fc-glycosylation regulates effector functions of human IgG subclasses, we cloned the antigen-binding region of the CD20-specific monoclonal antibody rituximab into IgG isotype expression vectors. We found that Fc-galactosylation enhances the efficacy of CD20-targeting complement-fixing antibodies for C1q binding and complement-mediated tumor cell lysis. Increased efficacies were restricted to IgG1 and IgG3 subclasses indicating that Fc-galactosylation alone is not sufficient for IgG2 and IgG4 to acquire complement-fixing properties. Addition of terminal galactose to the N-glycan specifically improved binding of C1q without changing antigen- and FcγRIIIa-binding affinities of IgG isotypes. These data indicate that Fc galactosylation can be harnessed to enhance the complement-activating properties of IgG1 and IgG3 antibodies.

## Introduction

Immunoglobulin gamma (IgG) antibodies are preeminent effector proteins of the immune system. The bimodal architecture of IgG allows for simultaneous recognition of antigen (by the antigen-binding fragment, Fab) and the initiation of IgG effector functions such as recruitment and activation of leukocytes and antibody-dependent cell-mediated cytotoxicity (ADCC) through interaction with FcγRs as well as complement activation through binding of the constant, crystallizable fragment (Fc) binding of C1q. Human IgG is subdivided in the four isotypes IgG1 to IgG4, numbered based on their abundance in serum (1). Despite being more than 90% identical in amino acid sequence, IgG isotypes differ in key functional regions responsible for flexibility, FcγR binding, and complement fixation (2).

All IgG subclasses contain a highly conserved asparagine-linked (N-)oligosaccharide located in the CH2 domain of the Fc region. Presence of this glycan serves important functions in protein folding and post-translational quality control mechanisms and is essential for antibody-mediated effector functions (3, 4). The biantennary core glycan structure, which is composed of two N-acetylglucosamines (GlcNAc) and three mannose residues, can be further decorated with fucose, bisecting GlcNAc and terminal GlcNAc, galactose, and sialic acid. The highest degree of variability among IgG antibodies stems from the presence or absence of galactose with around 40% containing one galactose, 20–40% two galactoses, and the remainder none (5–7).

Presence or absence of distinct N-glycan residues such as fucose and sialic acid can dramatically alter pro- and anti-inflammatory IgG activities. Removal of the fucose residue from human IgG1 increases antibody-dependent cell-mediated cytotoxicity (ADCC) through improved affinity for Fcγ receptor IIIa (FcγRIIIa) (8, 9). We have previously shown that IgG Fc sialylation of human monoclonal IgG1 molecules impairs their efficacy to induce complement-dependent cytotoxicity (CDC) (10). Here, we determined whether Fc galactosylation of human IgG isotypes (IgG1–4) regulates antibody effector functions.

## Results

### Generation of Degalactosylated and Galactosylated IgG Isotypes Targeting CD20

**Figure 1:**
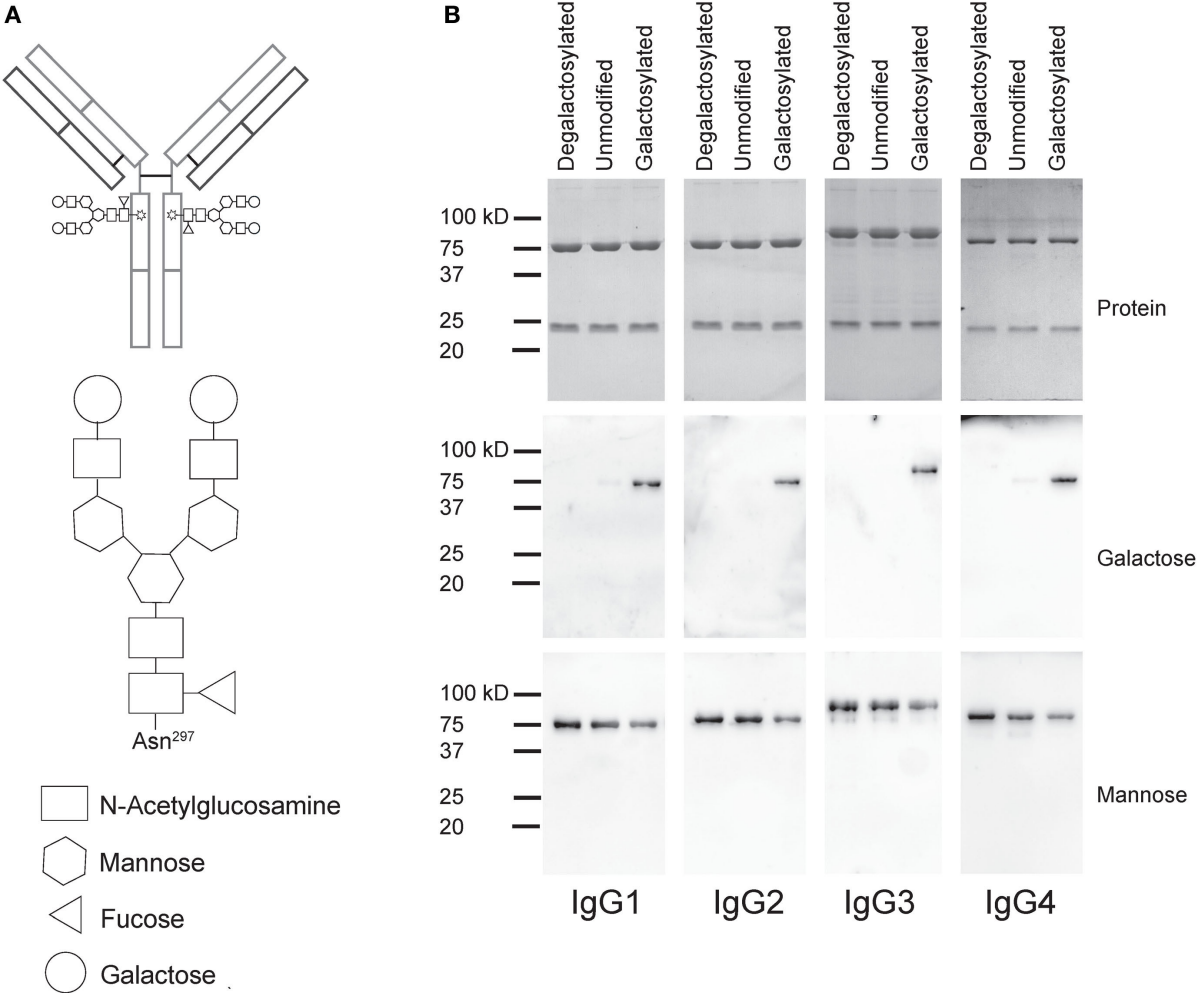
Generation of degalactosylated and galactosylated immunoglobulin gamma (IgG) isotypes derived from rituximab. **(A)** Schematic depiction of an IgG antibody with its two Fc N-glycans (up) and detailed glycan composition of a fully galactosylated IgG-Fc N-glycan. **(B)** Gel electrophoresis and coomassie staining to detect total protein showing heavy and light chains of antibodies (up) and immunoblots using galactose- (*Erythrina Cristagalli* lectin) or mannose- (*Lens Culinaris* agglutinin) specific lectins.

To test the impact of Fc galactosylation of human IgG isotypes on target cell depletion, we first cloned the sequence encoding the heavy chain (HC) and light chain (LC) antigen-binding regions of the monoclonal CD20-targeting antibody rituximab into human IgG1–4 HC and kappa LC expression vectors. Plasmids were cotransfected in HKB11 cells and antibodies were purified from the supernatant using protein G columns. Next, we either treated the antibodies with recombinant galactosidase to completely remove galactose or with β-1,4-galactosyltransferase in the presence of uridine diphosphate (UDP-) galactose to obtain galactosylated antibodies. After repurification, antibodies were analyzed for purity and integrity by gel electrophoresis and immunoblotting with the galactose-specific *Erythrina Cristagalli* lectin to confirm the successful generation of glycovariants (Figure 1).

### Addition or Removal of IgG-Fc Galactose Does Not Affect Antigen Binding

**Figure 2:**
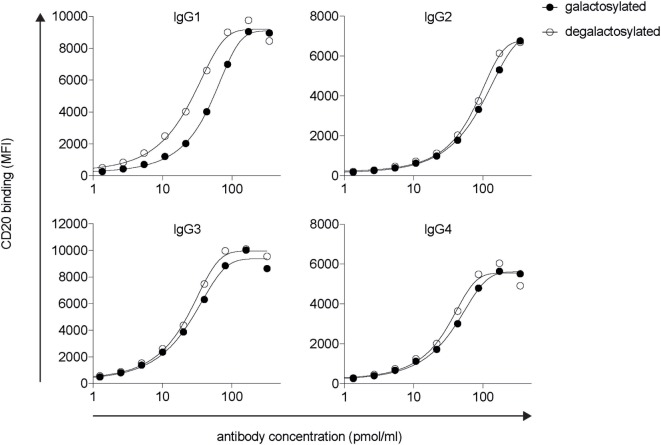
Fc-galactosylation does not increase CD20 binding of rituximab-derived IgG isotypes. Target antigen recognition by galactosylated and degalactosylated human IgG1–4 isotypes specific for CD20 analyzed by flow cytometry *via* titration on CD20^+^ Raji cells. MFI, median fluorescence intensity; IgG, immunoglobulin gamma.

Modifications in the Fc domain may change the structural properties of the antibody, potentially leading to changes in its antigen-binding region. To determine antigen-binding affinities of IgG isotype glycovariants, we titrated the antibodies on CD20 expressing human Raji-Burkitt’s lymphoma cells and analyzed binding by flow cytometry (Figure 2). For each isotype, galactosylated and degalactosylated glycovariants did not differ in their antigen-binding characteristics.

### IgG-Fc Galactosylation of IgG1 and IgG3 Isotypes Increases C1q Binding and Complement-Dependent Cytotoxicity

**Figure 3:**
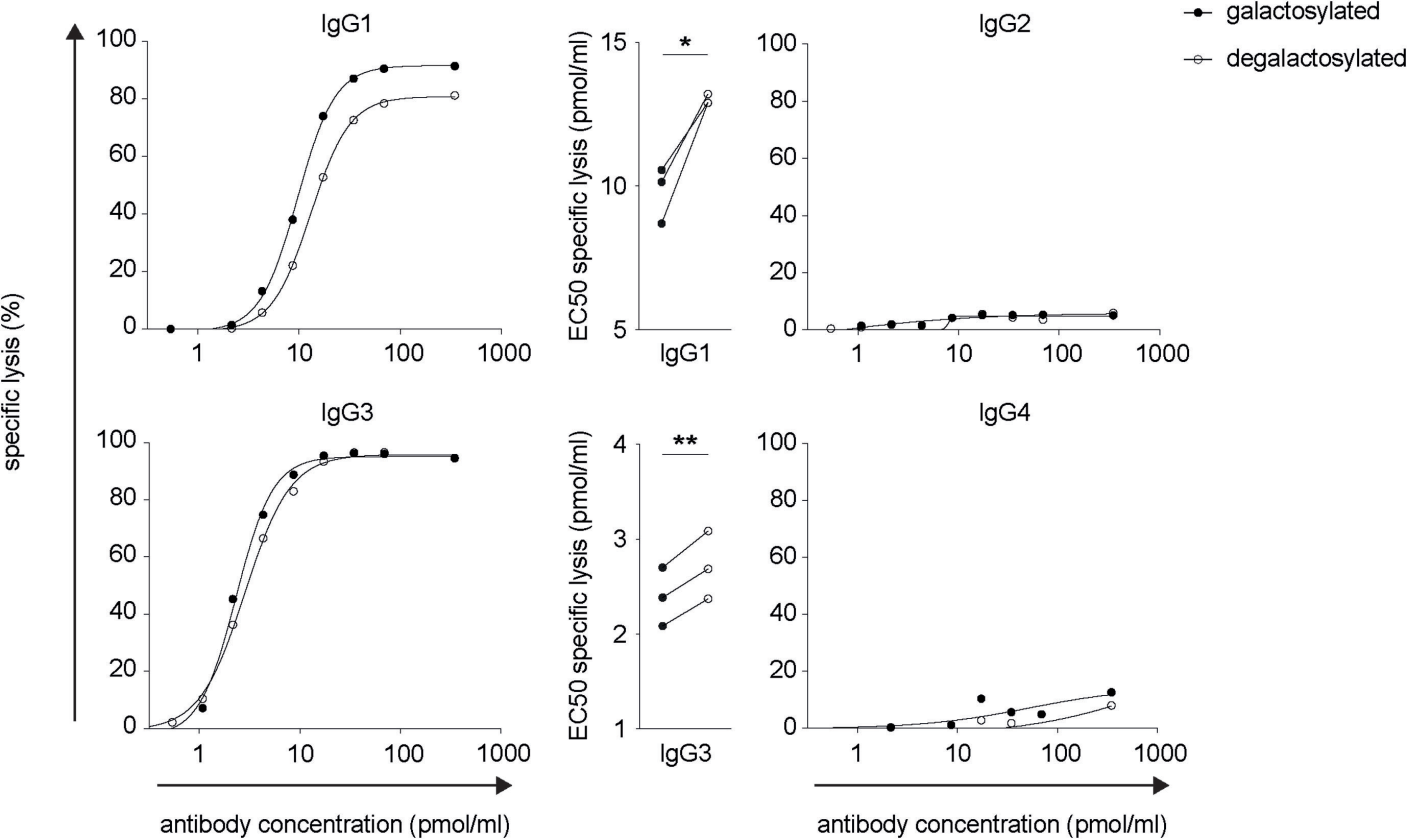
Increased CDC of rituximab-derived immunoglobulin gamma 1 (IgG1) and IgG3 but not IgG2 and IgG4 upon Fc-galactosylation. Complement-dependent lysis of CD20^+^ target cells in the presence of galactosylated or degalactosylated human anti-CD20 IgG isotypes. Exemplary lysis curves and EC50 values of three independent experiments are shown. Statistical analysis: paired two-tailed Student’s *t*-test *p < 0.05, **p < 0.01.

**Figure 4:**
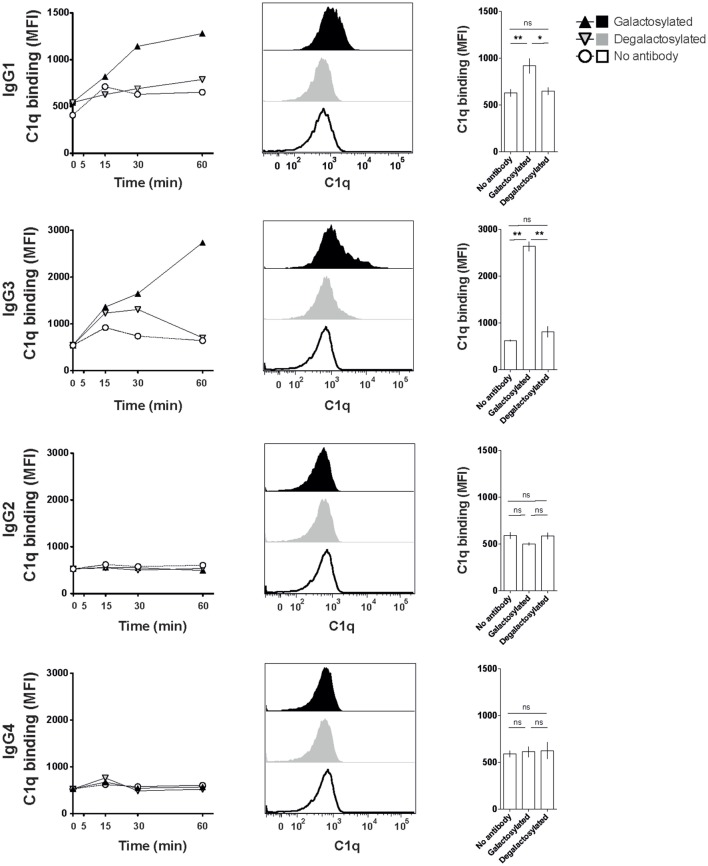
Increased C1q binding of Fc-galactosylated rituximab-derived immunoglobulin gamma 1 (IgG1) and IgG3. Kinetic of C1q binding to CD20^+^ Raji cells in the presence of galactosylated or degalactosylated glycovariants of human IgG1–4. Exemplary C1q-binding curves (left), flow cytometry histograms of C1q binding after 60 min (center) and statistics at time point 60 min of at least three independent experiments. Statistical analysis: unpaired two-tailed Student’s *t*-test, mean ± SEM, ns, not significant, *p < 0.05, **p < 0.01, and ***p < 0.001. MFI, median fluorescence intensity.

The efficacy of IgG isotype-derived glycovariants to induce complement-dependent cytotoxicity (CDC) was determined in Burkitt’s lymphoma-derived Raji cells in the presence of active complement (human serum). Rituximab-derived IgG3 glycovariants showed the highest efficacy for CDC, followed by IgG1 (Figure 3). Glycovariants of IgG2 and IgG4 isotypes did not induce CDC. Galactosylation increased CDC mediated by both IgG1 (26% reduction of EC50) and IgG3 (13% reduction of EC50) but did not provide IgG2 and IgG4 with *de novo* ability to lyse target cells (Figure 3). To investigate the mechanism by which Fc-galactosylation impacts CDC, we determined the C1q binding affinities and kinetics of galactosylated and degalactosylated antibody variants (10). Incubation of CD20-expressing Raji cells in the presence of human serum depleted for C5, an essential component of the complement cascade, which allows to analyze the binding of members of the complement cascade to target cells while preventing cell lysis (10), led to rapid binding of C1q (Figure 4). Fc-galactosylation substantially enhanced the antibodies’ capacity to bind C1q for IgG1 and IgG3 isotypes (Figure 4). These data indicate that the addition of terminal galactose to the Fc-glycan enhances cell-depleting efficacies of human IgG1 and IgG3 isotypes through increased C1q binding.

### Fc-Galactosylation Does Not Increase IgG:FcγRIIIa-Binding Affinities

**Figure 5:**
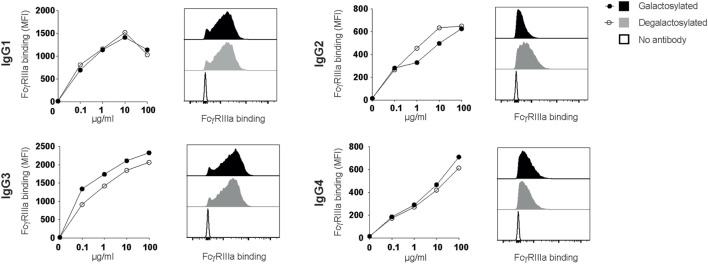
Immunoglobulin gamma (IgG) binding to FcγRIIIa does not require Fc-galactosylation. Binding of galactosylated and degalactosylated IgG isotypes to CHO cells transfected with FcγRIIIa was analyzed by flow cytometry. Binding curves and flow cytometry histograms at 10 µg/ml are shown.

Rituximab depletes B cells through a combination of CDC and antibody-dependent cell-mediated cytotoxicity (ADCC), which requires antibody binding to the human-activating FcγRIIIa (CD16) (10, 11). Absence of the IgG-Fc core fucose increases binding to FcγRIIIa (8, 12), a finding increasingly being used to improve the efficacy of therapeutic antibodies (13–15). Two studies reported that Fc-galactosylation results in a slight, albeit not statistically significant increase in FcγRIIIa-binding affinity and ADCC activity (16, 17). A more recent study confirmed that afucosylated glycoforms show higher binding affinities for FcγRIIIa, while Fc-galactosylation did not significantly impact FcγRIIIa binding (18). To investigate whether Fc-galactosylation of IgG isotype glycovariants increases binding affinities of IgG isotypes for FcγRIIIa, thereby potentially enhancing the antibodies’ ability to induce ADCC, we titrated IgG glycovariants onto CHO-derived cell lines recombinantly expressing FcγRIIIa. FcγRIIIa binding was strongest for IgG1 and IgG3 isotypes (Figure 5). IgG-Fc galactosylation showed, however, no effect on FcγRIIIa-binding affinities for any of the IgG isotypes tested (Figure 5). Thus, while increasing the antibodies’ affinity for C1q binding and their efficacy to induce CDC, Fc-galactosylation did not change the affinity of human IgG isotypes for FcγRIIIa which, upon ligation, mediates ADCC.

## Discussion

Initiation of the classical complement cascade trough binding of C1q is a potent proinflammatory mechanism by which IgG antibodies trigger immune responses during infection and its deregulation causes tissue damage in a wide array of human inflammatory, degenerative, and autoimmune diseases (19). Our study shows that Fc-galactosylation enhances the efficacy of complement-fixing IgG isotypes to induce CDC through improved binding of C1q.

The antibody-dependent classical complement pathway is initiated if the C1 complex, formed by the multimeric pattern recognition molecule C1q and the modular proteases C1r and C1s, docks on antigen-bound IgG (20). C1q binds to monomeric IgG with very low affinity but antigen-driven antibody clustering allows for the formation of IgG hexamers that bind C1q with high avidity and promote efficient complement activation (21). The N-glycan resides in the CH2 domain, which is required for C1q binding (22). Sites on the surface of human IgG1 that constitute the C1q-binding epicenter are conserved in human IgG isotypes that are deficient in C1q binding and it has therefore been suggested that the composition of the N-glycan might be critical for the antibodies’ conformation and its ability to bind C1q (23). Each biantennary oligosaccharide chain extends one arm toward the CH2–CH3 interface region and the other arm into the space between the CH2 domains, resulting in multiple interactions with the surface of the CH2 domain and the glycan of the opposing CH2 domain, respectively (4). Indeed, optimal C1 activation requires the presence of the Fc-glycan since C1q-mediated effector functions are compromised or lost in aglycosylated or deglycosylated IgGs (24–26). The effect of deglycosylation on reducing C1q binding has recently been attributed to its inhibition of IgG hexamerization *via* modulation of IgG Fc:Fc interactions rather than reduction of direct C1q-Fc-binding affinities (27). Based on these data and our results, we suggest that Fc-galactosylation modulates Fc:Fc interactions for antigen-bound IgG, thereby improving binding of C1q and increasing the antibodies’ ability to induce classical complement activation and CDC.

We systematically investigated whether Fc-galactosylation facilitates C1q binding and CDC effector functions across all human IgG isotypes. For murine IgG2b and IgG1, it has been demonstrated that addition of terminal galactose increases binding of C1q (28). While presence of terminal galactose enhanced complement activation by CD20 targeting, C1q-fixing human IgG1 and IgG3 isotypes, IgG2 and IgG4 remained deficient in initiating the classical complement cascade indicating that Fc-galactosylation alone is not sufficient for IgG2 and IgG4 to acquire complement-fixing properties.

Rituximab and therapeutic monoclonal antibodies (mAbs) that target tumor cells *via* ADCC or CDC are approved for the treatment of various cancers (29). B-cell depletion by CD20-targeting antibodies is also widely used for the treatment of autoimmune diseases (30). However, some patients do not sufficiently respond to rituximab therapy (31, 32) and improved versions of B cell depleting antibodies have been developed to increase ADCC activity and improve clinical efficacy (33, 34). Our data indicate that Fc-galactosylation, in addition to the established effect of defucosylation on ADCC, increases target cell cytotoxicity through enhancing CDC. Fc-galactosylation specifically improved binding of C1q to human IgG1 and IgG3 without changing antigen-binding affinities. Our data therefore indicate that Fc-galactosylation should be harnessed in glycoengineering therapeutic antibodies for effective antibody-dependent complement activation and target cell killing.

## Materials and Methods

### Buffers and Reagents

Human serum complement (Cat. No.: A100; Lot No.: 035997) and C5-depleted human serum (Cat. No.: A501; Lot No.: 031795) were purchased from Quidel^®^ Corporation. Accutase was purchased from StemCell Technologies Inc. (Cat. No.: 07920), Gibco™ RPMI-1640, HEPES (Cat. No.: 52400025), penicillin–streptomycin (P/S; 5,000 U/ml; Cat. No.: 15070063), LIVE/DEAD Fixable Aqua Dead Cell Stain Kit (Cat. No.: L34957), LIVE/DEAD Fixable Near-IR Dead Cell Stain Kit (Cat. No.: L10119), and TO-PRO-3 Stain (Cat. No.: T3605) were purchased from Invitrogen. Fetal calf serum (FCS) was purchased from Sigma-Aldrich (Cat. No.: F7524-500M; Lot No.: 051M3395) and from Bioswisstech (Cat. No.: S0615; Lot No.: 1047D). β1-4 galactosidase (Cat. No.: 345806-50MIU) and UDP-galactose (Cat. No.: 670111-50MG) were purchased from Merck Millipore Corporation, Calbiochem^®^. Protein-G sepharose was purchased from GE healthcare (Cat. No.: 17-0618-01). Biotinylated *Erythrina Cristagalli* lectin (Cat. No.: B-1145) and *Lens Culinaris* Agglutinin (Cat. No.: B-1045) were purchased from Reactolab. Phosphate-buffered saline (PBS; 2.68 mM KCl, 8 mM Na_2_HPO_4_, 1.8 mM KH_2_PO_4_, 137 mM NaCl, pH adjusted to 7.3) was produced in-house.

### Antibodies and Streptavidin

Monoclonal mouse anti-human C1q (Cat. No.: A201) and biotinylated anti-human C1q (Cat. No.: A700) were purchased from Quidel^®^ Corporation. Polyclonal rabbit anti-mouse IgG (H + L)-Alexa Fluor 488 (Cat. No.: A-11059) was purchased from Invitrogen. PE-conjugated streptavidin (Cat. No.: 405203) was purchased from Biolegend.

### CDC Assay

Raji cells were used as CD20^+^ target cells to assess CDC. Accordingly, 7 × 10^4^ Raji cells were cultivated in RPMI-1640 containing P/S (50 U/ml) in a humidified incubator (37°C, 5% CO_2_) in 96-well V-bottom plates. Cells were incubated with titrated concentrations of the respective galactosylated and degalactosylated anti-CD20 antibody isotypes. After 30 min, human serum complement was added to a final concentration of 5% and incubation was continued for another 12 h. Thereafter, cells were washed twice by adding 200 µl cold PBS and centrifugation at 400 × *g* and 4°C for 5 min. Cells were resuspended in cold PBS and TO-PRO3 stain (final concentration of 200 nM) was added to detect dead cells. All samples were analyzed on a BD FACSCanto-II using FACSDiva v6.1.3 software and FlowJo software v9.3.1 (Tree Star Inc.). Specific lysis was calculated as percent increase in dead cells compared to spontaneous lysis in the absence of an anti-CD20 antibody. EC50 values were calculated *via* a non-linear regression in GraphPad Prism 5.

### C1q-Binding Assay

A total of 2 × 10^5^ Raji cells were incubated with 10 µg/ml with galactosylated and degalactosylated anti-CD20 antibody isotypes in RPMI-1640 containing 1% P/S (50 U/ml) and 1% C5-depleted human serum for 5, 15, 30, or 60 min in a humidified incubator (37°C, 5% CO_2_). After incubation with the different anti-CD20 antibody isotypes, cells were washed twice with 200 µl cold PBS, and C1q binding was determined by adding biotinylated anti-C1q antibody (25 µg/ml in PBS) for 1 h on ice followed by washing twice with 200 µl cold PBS and incubation with PE-conjugated streptavidin (1:400 in PBS) and dead cell stain (LIVE/DEAD Fixable Aqua Dead Cell Stain Kit; 1:250 in PBS) for 20 min. After washing twice with 200 µl PBS, samples were analyzed on a BD FACSCanto-II using FACSDiva v6.1.3 software and FlowJo software v9.3.1 (Tree Star Inc.).

### CD20-Target-Binding Assay

Raji cells were maintained in RPMI-1640 containing P/S (50 U/ml) and 10% FCS in a humidified incubator (37°C, 5% CO_2_). 1.2 × 10^5^ Raji cells and anti-CD20 antibody isotypes and glycovariants were incubated for 25 min on ice. Dead cells were excluded using LIVE/DEAD Fixable Near-IR Dead Cell Stain Kit. Cells were washed twice with 200 µl PBS and anti–mouse IgG (H + L)-Alexa Fluor 488 was added and incubated for 20 min on ice followed by two washing steps with 200 µl cold PBS. All samples were analyzed on a BD FACSCanto-II using FACSDiva v6.1.3 software and FlowJo software v9.3.1 (Tree Star Inc.).

### FcγR-Binding Assay

Human FcγRIIIa-expressing CHO cells (35) were kindly provided by Falk Nimmerjahn (Department of Biology, University of Erlangen-Nürnberg, Germany). Cells were maintained in RPMI-1640 containing P/S (50 U/ml) and 10% FCS in a humidified incubator (37°C, 5% CO_2_). To detect binding of anti-CD20 antibodies, cells were detached using accutase, washed with cold PBS, and 2 × 10^5^ cells were incubated on ice for 30 min with anti-CD20 antibodies. After incubation, cells were washed twice with 200 µl cold PBS and anti-mouse IgG (H + L)-Alexa Fluor 488 was added for 20 min on ice. All samples were washed twice with 200 µl cold PBS and analyzed on a BD FACSCanto-II using FACSDiva v6.1.3 software and FlowJo software v9.3.1 (Tree Star Inc.).

### Generation of Recombinant Monoclonal Anti-CD20 IgG Isotypes

The DNA encoding for the N terminal region of the light chain (amino acids QIVLS until KLEIK) and heavy chain (amino acids QVQLQ until TVSAA) of rituximab (www.drugbank.ca; accession number DB00073) was synthesized by Invitrogen GeneArt gene synthesis. The N terminal region of the light chain was cloned into the multiple cloning site (MCS) of Igκ-AbVec (NCBI GenBank accession number FJ475056.1) using *Age*I and *Bsi*WI as previously described (36). To obtain human IgG-1, -2, -3 and -4 heavy chain expression vectors IgG-AbVec (NCBI GenBank accession number FJ475055.1) (36) was modified to encode for human IgG1 (allotype G1m17,1), IgG2 (allotype G2m), IgG3 (allotype G3m), and IgG4 (kindly provided by Lars Hangartner, the Scripps Research Institute, Department of Immunology and Microbial Science, La Jolla, CA, USA) (2, 37, 38). The N terminal region of the rituximab heavy chain was cloned into the MCS using *Age*I and *Sal*I as previously described (36). Recombinant monoclonal antibodies were expressed and purified as previously described (10, 32, 39). Briefly, heavy and light chain expression plasmids were cotransfected into a human B cell–epithelial cell fusion cell line (HKB11) supporting high-level recombinant protein expression (40) using calcium phosphate-mediated transfection. After 6 days, antibodies were purified from cell culture supernatants by binding to a protein G column and elution with 0.1 M glycine (pH 2) followed by neutralization with 1 M Tris pH 8.8 and dialysis to PBS. Antibody purity and integrity were confirmed by polyacrylamide gel electrophoresis, coomassie brilliant blue staining and Western blotting with isotype-specific monoclonal antibodies.

### Generation of Galactosylated and Degalactosylated Antibody Glycovariants

Antibody glycovariants were generated as previously described (10, 39). For removal of galactose, antibodies were dialyzed to 50 mM sodium phosphate buffer pH 6.0, 60 mU β1–4 galactosidase was added per mg of antibody and the reaction was incubated for 6 h at room temperature followed by 1 h at 37°C. To add galactose, antibodies were dialyzed to 0.2 M MES pH 6.5 followed by the addition of 10 mM MnCl_2_, UDP-galactose, 0.02% NaN_3_, and 5 µg recombinant β1–4 galactosyltransferase [produced in-house (39)]. The reaction was incubated at 37°C for 48 h. Finally, all antibodies were centrifuged for 2 h at 4°C and >20,000 *× g* to remove aggregates, repurified by gravity-flow protein-G sepharose columns, and dialyzed to PBS. Successful degalactosylation and galactosylation were monitored by lectin-blotting using the galactose-specific lectin from *Erythrina Cristagalli*.

### Statistics

A *p*-value of 0.05 or less was defined as statistically significant. For all analyses, Prism software, version 5 (GraphPad Software, Inc.) was used. Specific statistical tests are indicated in the respective figure legends.

## Author Contributions

IQ and JL conceived the study. JL designed, BP, CK, and IQ designed and performed experiments. PW performed experiments. All the authors cowrote and approved the manuscript.

## Conflict of Interest Statement

The authors declare that the research was conducted in the absence of any commercial or financial relationships that could be construed as a potential conflict of interest.
